# Measuring the Impact: Magnetic Resonance Imaging Response of Sacroiliac Joints to Tumor Necrosis Factor Inhibitors in Youth With Axial Disease

**DOI:** 10.1002/art.70050

**Published:** 2026-03-19

**Authors:** Timothy G. Brandon, Rui Xiao, Daniel J. Lovell, Edward Oberle, Matthew L. Stoll, Nancy A. Chauvin, Michael L. Francavilla, Walter P. Maksymowych, Pamela F. Weiss

**Affiliations:** ^1^ Department of Pediatrics, Division of Rheumatology and Clinical Futures Children's Hospital of Philadelphia Philadelphia Pennsylvania; ^2^ Department of Biostatistics, Epidemiology and Informatics Perelman School of Medicine, University of Pennsylvania Philadelphia; ^3^ Division of Rheumatology Cincinnati Children's Hospital Medical Center, University of Cincinnati Cincinnati Ohio; ^4^ Department of Pediatrics, Division of Rheumatology Nationwide Children's Hospital and The Ohio State University Columbus Ohio; ^5^ Department of Pediatrics University of Alabama at Birmingham Birmingham Alabama; ^6^ The Imaging Institute, The Cleveland Clinic Cleveland Ohio; ^7^ Department of Radiology Whiddon College of Medicine, University of South Alabama Mobile Alabama; ^8^ Department of Medicine University of Alberta Edmonton Alberta Canada; ^9^ CARE Arthritis Edmonton Alberta Canada; ^10^ Center for Clinical Epidemiology and Biostatistics, Perelman School of Medicine, University of Pennsylvania Philadelphia Pennsylvania

## Abstract

**Objective:**

To evaluate the timeline for resolution of sacroiliac joint (SIJ) inflammation, changes in structural lesions, and their correlation with patient‐reported outcomes (PROs) in youth with axial juvenile spondyloarthritis (axJSpA) initiating tumor necrosis factor inhibitor (TNFi).

**Methods:**

This prospective, multicenter study included youth aged 8 to 18 years with a clinical diagnosis of axJSpA starting TNFi. Assessments were conducted at baseline and 12 weeks, including clinical evaluation, magnetic resonance imaging (MRI), and PROs. Participants with persistent SIJ inflammation at 12 weeks were reassessed at 24 weeks. Three blinded reviewers evaluated MRIs using Spondyloarthritis Research Consortium of Canada SIJ inflammation scores (SIS) and SIJ structural scores.

**Results:**

Of 75 enrolled participants, 73 completed baseline visits, and 62 had MRI‐confirmed axJSpA. Fifty‐seven completed a 12‐week follow‐up; 89% (51 of 57) showed SIS improvement, and 63% (36 of 57) achieved inflammation resolution (SIS <2). Median SIS change from baseline to 12 weeks was −8 (interquartile range: −18 to −3). Among those with persistent inflammation at 12 weeks (n = 26), 85% reported at least moderate clinical improvement. At 24 weeks, 56% (14 of 25) had ongoing inflammation. A total of 84% of SIS improvement occurred within the first 12 weeks. In patients with at least two scans, structural lesion scores decreased, increased, or stayed the same from baseline to the 12‐week scan for erosions (58%/25%/18%), sclerosis (21%/9%/70%), fat metaplasia (0%/30%/70%), and backfill (4%/28%/68%).

**Conclusion:**

Most participants showed early imaging response to TNFi, with most improvement occurring within 12 weeks. Despite residual inflammation persisting in nearly half of patients, most reported symptom improvement, underscoring both the rapid impact of TNFi and the heterogeneity of treatment effects.

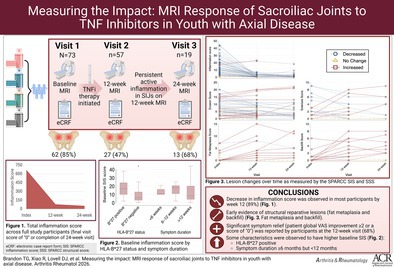

## Graphical abstract

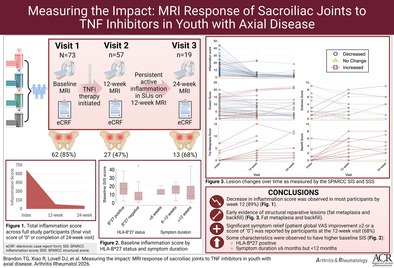

## INTRODUCTION

Juvenile spondyloarthritis (JSpA), classified under the International League of Associations for Rheumatology (ILAR) criteria for juvenile idiopathic arthritis (JIA) as either psoriatic arthritis or enthesitis‐related arthritis, accounts for approximately 15% to 25% of pediatric arthritis cases. Approximately 20% of youth have axial involvement (axial JSpA [axJSpA]) at the time of presentation.^1^ Although evidence from clinical trials in youth remains scarce, tumor necrosis factor inhibitors (TNFi) are recommended as a first‐line biologic therapy for sacroiliitis, following nonsteroidal anti‐inflammatory drugs (NSAIDs), in the 2019 American College of Rheumatology/Arthritis Foundation treatment guidelines for juvenile arthritis.^2^

Recent advances have begun to address critical gaps in the field that have limited research and clinical trial design in this population. The first classification criteria for axial disease in youth with JSpA were developed and validated,^3^ enabling more consistent patient identification and facilitating the design and conduct of novel treatment and outcomes studies. Standardized, data‐driven magnetic resonance imaging (MRI) definitions of active and structural sacroiliac joint (SIJ) lesions in youth with axial disease were also developed.^4^ These MRI definitions were leveraged in the axJSpA classification criteria to define imaging consistent with unequivocal inflammation and structural damage. These MRI definitions can also be leveraged for outcome assessment and entry criteria for future studies. A core set of responsive and discriminatory disease activity and outcome measures for axJSpA trials has also been defined.^5^ As novel therapies such as interleukin‐17, JAK‐STAT, and TYK‐2 inhibitors demonstrate efficacy in adults with axial SpA, understanding the SIJ imaging response to TNFi in youth is critical. TNFi remain the most widely used biologics in pediatric sacroiliitis, and characterizing their effects provides a benchmark for comparison with emerging therapies.

The objectives of this study were to evaluate the timeline of SIJ inflammation resolution, changes in structural lesions, and their relationship to patient‐reported outcomes (PROs) in youth with incident axJSpA initiating TNFi therapy. These findings have significant implications for optimizing clinical monitoring, informing expectations of therapeutic response, and guiding future JIA trial design in pediatric rheumatology.

## PATIENTS AND METHODS

### Human participants protection

The protocol for this prospective study was reviewed and approved by the Institutional Review Boards at Children's Hospital of Philadelphia, Nationwide Children's Hospital, University of Alabama at Birmingham, and Cincinnati Children's Hospital Medical Center. HIPAA (Health Insurance Portability and Accountability Act) authorization and informed consent or parental permission plus assent was obtained from all participants.

### Participants

This is a secondary analysis of a previously reported multicenter, prospective study conducted between 2019 and 2024 at four pediatric hospitals.^5^ The study enrolled youth aged 8 to 18 years with JSpA and axial disease (axJSpA) who met all of the following inclusion criteria: (1) fulfillment of ILAR criteria for enthesitis‐related arthritis or psoriatic arthritis^6^ or the European SpA Study Group criteria,^7^ (2) no previous treatment with a biologic agent (biologic‐naïve), (3) physician‐confirmed axial arthritis based on clinical or imaging findings, and (4) initiation of TNFi deemed necessary by primary rheumatologist as part of routine clinical care. Concurrent or previous treatment with a conventional synthetic disease‐modifying antirheumatic drug (csDMARD) was recorded but not included as part of the eligibility criteria for participation.

### Assessments

Participants underwent baseline and 12‐week assessments, including questionnaires, clinical examination, and MRI. All participants completed the baseline questionnaires and MRI scan before or up to two weeks after initiation of TNFi therapy. Follow‐up visits were conducted 12 ± 6 weeks from the baseline MRI scan or first TNFi dose, whichever came second, but no more than 24 weeks after the baseline MRI scan to accommodate participants who encountered issues obtaining medication. Participants with persistent SIJ inflammation as determined by a single central radiologist at 12 weeks were invited to undergo an additional MRI scan and questionnaires at 24 weeks (12 ± 6 weeks after the 12‐week visit). Imaging was assessed by three blinded, independent reviewers, with data aggregated without consideration of time point. PROs included Patient‐Reported Outcomes Measurement Information System (PROMIS) pain interference and global health scores, neck/back/hip pain self‐assessments, and an evaluation of overall disease activity (improved, stable, or worsened). All raw scores generated from PROMIS instruments are translated into standardized T‐scores with a population mean of 50 and a SD of 10. Higher scores in a domain represent more of the trait being measured; higher T‐scores indicate a worse outcome in pain interference; lower T‐scores indicate a worse outcome in global health.

#### Imaging

MRI sequences included a coronal oblique STIR, a coronal oblique T1‐weighted turbo spin echo, and an axial T2‐weighted turbo spin echo with fat suppression. A centralized imaging panel of three experienced imaging experts (WPM, NC, MF) evaluated the scans. De‐identified Digital Imaging and Communications in Medicine images were reviewed using dedicated scoring modules that are available at carearthritis.com. MRIs were assessed for inflammatory lesions of the SIJs using the Spondyloarthritis Research Consortium of Canada (SPARCC) SIJ inflammation score (SIS),^8^ which evaluates the depth and intensity of bone marrow edema across both SIJ. SIS range from 0 to 72, with higher scores indicating greater inflammation; this tool is validated in pediatric populations.^9^, ^10^, ^11^ Lesion score ratings from the three imaging experts were averaged to generate an average score per lesion and participant. The published minimally important change in adults is −2.5.^8^ An SIS <2 was selected to represent negative MRI scans during analysis because it was the value most aligned with the 2009 Assessment of Spondyloarthritis international Society (ASAS)/Outcomes Measures in Rheumatology (OMERACT) definition for active sacroiliitis.^12^, ^13^

Structural changes were evaluated using the SPARCC sacroiliac structural score (SSS),^14^ which assesses fat metaplasia, erosion, fat metaplasia in an erosion cavity (backfill), and ankylosis. For pediatric cases, we also included sclerosis. Components were scored as follows: 0 to 40 for sclerosis, fat metaplasia, and erosion and 0 to 20 for fat within an erosion cavity and ankylosis. Higher numbers indicate more structural changes; this measure is validated in youth.^15^

### Analysis

Demographics, clinical features, and measurements collected on the study cohort were summarized by median and interquartile range (IQR). Differences in SIS and structural change(s) by sex, overweight status (dichotomized at body mass index [BMI] z‐score ≥85th percentile), HLA‐B27 status, presence of elevated markers of inflammation, and symptom duration were assessed using the Wilcoxon Mann‐Whitney (for medians) and chi‐square (for proportions) tests as appropriate. Age and symptom duration associations with baseline imaging findings were evaluated with linear or logistic regression as appropriate. Logistic regression analysis examined associations between participant characteristics and improvement or no change/worsening in the SIS from baseline to week 12 and achievement of an SIS ≤2 at weeks 12 and 24. Associations between imaging scores and PROs were analyzed using Spearman's correlation. Interpretation of the Spearman correlation coefficients follows the same conventional interpretations for Pearson correlation coefficients: – ≤|0.1| negligible, <|0.4| weak, <|0.7| moderate, <|0.9| strong, and ≥|0.9| very strong.^16^ The data that support the findings of this study are available from the corresponding author on reasonable request.

## RESULTS

### Participants

Seventy‐five participants were enrolled, 73 (97.3%) completed the baseline visit, and 71 (94.6%) had an MRI available for detailed scoring by the central imaging team. Of the 73 completing the baseline visit, 62 (84.9%) had MRI findings typical of axJSpA, and 57 of 62 (91.9%) completed the 12‐week visit. Participant demographics and clinical characteristics are shown in Table 1. Symptom duration at baseline was at least 6 weeks and at least 12 weeks for 79.5% and 61.6% of participants, respectively. Adalimumab (n = 58, 80.6%) and etanercept (n = 11, 15.3%) were the most common TNFi therapies started. No participants discontinued or changed TNFi during the study. Of the 62 participants with imaging typical of axJSpA, findings were bilateral in 27 (43.6%). Of the 86 SIJs affected across the 62 participants, 20 (23.2%), 18 (20.9%), and 48 (55.8%) had changes in the ilium, sacrum, or both, respectively.

**Table 1:** Participant characteristics at baseline*

Baseline characteristics	All participants		Participants with axJSpA MRI findings		Participants with ≥2 MRI scans	
	N	Median (IQR) or n (%)	N	Medain (IQR) or n (%)	N	Median (IQR) or n (%)
Criteria fulfilled						
ESSG, n (%)	73	57 (78.1)	62	52 (83.9)	57	48 (84.2)
ILAR ERA, n (%)	73	60 (82.2)	62	53 (85.5)	57	49 (86.0)
ILAR PsA, n (%)	73	4 (5.5)	62	3 (4.8)	57	3 (5.3)
Age at baseline, mean (IQR), years	73	15.5 (13.6–17.0)	62	15.5 (13.5–17.0)	57	15.3 (13.5–16.9)
Male, n (%)	73	45 (61.6)	62	41 (66.1)	57	39 (68.4)
Race, n (%)	73		62		57	
White		53 (72.6)		44 (71.0)		42 (73.7)
Asian		6 (8.2)		6 (9.7)		5 (8.8)
Black or African American		5 (6.9)		4 (6.5)		3 (5.3)
Other		4 (5.5)		4 (6.5)		3 (5.3)
Unknown		3 (4.1)		2 (3.2)		2 (3.5)
Native Hawaiian or other Pacific Islander		1 (1.4)		1 (1.6)		1 (1.8)
More than one race		1 (1.4)		1 (1.6)		1 (1.8)
Ethnicity, not Hispanic or Latino/a, n (%)	70	67 (95.7)	60	57 (95.0)	55	53 (96.4)
HLA‐B*27+, n (%)	66	30 (45.5)	57	25 (43.9)	53	22 (41.5)
Family history of SpA, n (%)	68	9 (13.2)	58	7 (12.1)	54	5 (9.3)
csDMARDs, n (%)						
Sulfasalazine	73	2 (2.7)	62	2 (3.2)	57	2 (3.5)
Methotrexate (any)	73	17 (23.3)	62	17 (27.4)	57	17 (29.8)
≤10 mg per week^a^	73	13 (17.8)	62	13 (21.0)	57	13 (22.8)
>10 mg per week	73	4 (5.5)	62	4 (6.5)	57	4 (7.0)
Symptom duration (≥12 weeks), n (%)	73	45 (61.6)	62	39 (62.9)	57	38 (66.7)
AJC ≥3, n (%)	73	11 (15.1)	62	7 (11.3)	57	7 (12.3)
Peripheral arthritis, n (%)	73	20 (27.4)	62	16 (25.8)	57	16 (28.1)
Enthesitis, n (%)	73	40 (54.8)	62	34 (54.8)	57	31 (54.4)
Psoriasis, n (%)	73	6 (8.2)	62	6 (9.7)	57	6 (10.5)
Inflammatory bowel disease, n (%)	73	9 (12.3)	62	9 (14.5)	57	9 (15.8)
SPARCC SIS, mean (IQR)	71	10 (3–19)	62	10.5 (4–20)	57	11 (5–20)
SPARCC SSS: lesion present/absent, n (%)	71		62		57	
Sclerosis		22 (31.0)		22 (35.5)		21 (36.8)
Erosion		56 (78.9)		54 (87.1)		51 (89.5)
Fat in an erosion cavity		8 (11.3)		8 (12.9)		6 (10.5)
Fat metaplasia		7 (9.9)		7 (11.3)		6 (10.5)
Ankylosis		2 (2.8)		2 (3.2)		2 (3.5)

* AJC, active joint count; axJSpA, axial juvenile spondyloarthritis; csDMARD, conventional synthetic disease‐modifying antirheumatic drug; ERA, enthesitis‐related arthritis; ESSG, European Spondyloarthritis Study Group; ILAR, International League of Associations for Rheumatology; IQR, interquartile range; MRI, magnetic resonance imaging; PsA, psoriatic arthritis; SIS, sacroiliac joint inflammation score; SpA, spondyloarthritis; SPARCC, Spondyloarthritis Research Consortium of Canada; SSS, sacroiliac joint structural score.

^a^ ≤10 mg per week of methotrexate generally prescribed to help prevent human anti‐human antibody formation.

### Inflammation

**Figure 1:**
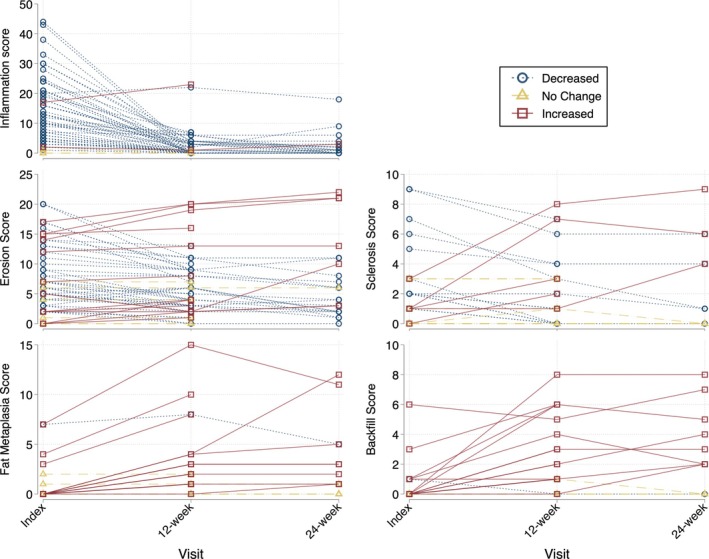
Change in lesions over time. The SPARCC SSS^14^ assesses a spectrum of structural lesions of the sacroiliac joint on MRI, including fat metaplasia, erosion, backfill, and ankylosis; for pediatric cases we also included sclerosis. These components are scored 0 to 20 (backfill and ankylosis) or 0 to 40 (sclerosis, fat metaplasia, erosion), with higher numbers indicative of more structural changes; this measure is validated in youth. Participants are color‐coded based on lesion score change from baseline to final visit. MRI, magnetic resonance imaging; SPARCC, Spondyloarthritis Research Consortium of Canada; SSS, sacroiliac structural score.

A total of 84.9% (62 of 73) had detectable subchondral SIJ inflammation at baseline. The median baseline SIS was 10 (IQR 3 to 19). Among the 57 participants with at least two MRIs, 51 (89.5%) demonstrated improvement in the SIS by week 12 of TNFi therapy, with a median change of −8 (IQR −18 to −3) (Figure 1). A total of 63% achieved resolution of inflammation, which was defined as an SIS <2 at week 12. A small number of participants (n = 4) had no change, and two showed worsening in the SIS over the same period.

Baseline SIS was significantly higher between participants who were HLA‐B27 positive versus negative (14 vs 6; *P* = 0.02) and highest for those with symptom duration of at least 6 weeks but under 12 weeks compared with at least 12 weeks and under 6 weeks (18 vs 7 vs 4; *P* = 0.02). There were no significant differences in baseline SIS by age, sex, overweight status, or elevation of markers of inflammation. SIS at the 12‐week visit were statistically improved in boys versus girls (−10 vs 5.5; *P* = 0.047) and in HLA‐B27 positive versus negative participants (−15.5 vs −4; *P* < 0.001). There were no statistically significant differences in change scores based on age at MRI, normal versus overweight/obese participants, normal versus elevated markers of inflammation, or symptom duration at baseline.

Of the 27 participants with residual inflammation at week 12, 19 completed the week 24 scan, and 68.4% (13 of 19) continued to show inflammation. Univariable logistic regression did not reveal any statistically significant predictors of SIS ≥2 at week 12 among sex, HLA‐B27 status, or the following baseline measures: BMI category, SIS, disease duration, CRP, ESR, or age. The median SIS change from week 12 to 24 was −2 (IQR −3 to 0). A total of 83% of the total SIS improvement across all participants occurred within the first 12 weeks of therapy.

### Structural lesions

At baseline, 78.9% (56 of 71) of participants had at least one structural lesion on MRI, with erosion being the most common finding (all 56 participants with >0 structural lesions had at least one erosion). Differences in the proportions of structural changes were not statistically significant for any lesion type by sex, overweight status, HLA‐B27 positivity, or elevated markers of inflammation, except for erosions, which were more frequent in patients with elevated markers of inflammation (median 6, IQR 3–14) compared with those with normal markers (median 5, IQR 0–7).

Figure 1 shows changes over time in the structural lesions. Among the 57 participants who underwent at least two MRIs, erosion and sclerosis scores decreased at week 12 in 57.9% (n = 33) and 21.1% (n = 12), respectively, with median changes of −3 (IQR −4 to −2) for erosion and −2 (IQR −2.5 to −1) for sclerosis. A small proportion had no change in the erosion score (17.5%), whereas most had no change in the sclerosis score (70.2%). Erosion scores worsened in 24.6% of participants and 8.8% for sclerosis, with median changes of 2 (IQR 1–3) and 2 (IQR 2 –5), respectively. Of the 14 participants with a worse erosion score at week 12, 10 (71.4%) also had evidence of continued inflammation as reflected by an SIS of ≥2. Of the 12 participants with improvement in the sclerosis score at week 12, 100% had improvement in the SIS. Change in the erosion score and sclerosis score were not correlated (r_s_ = 0.15).

Structural lesions of fat metaplasia and backfill, which are thought to reflect a tissue response to the resolution of inflammation, also showed changes over time. By week 12, 29.8% (n = 17) of participants had increased fat metaplasia scores, and 28.1% (n = 16) had increased backfill scores, with median changes of 2 (IQR 1–4) and 2 (IQR 1–3), respectively. Of these participants with improvement in the fat metaplasia and backfill scores at week 12, 100% had improvement in the SIS.

Scores remained unchanged in 70.2% (n = 40) for fat metaplasia and 68.4% (n = 39) for backfill. A decrease in backfill score was observed in only two participants, whereas no participants showed a reduction in the fat metaplasia score.

### Patient‐reported responses and association with imaging responses

**Figure 2:**
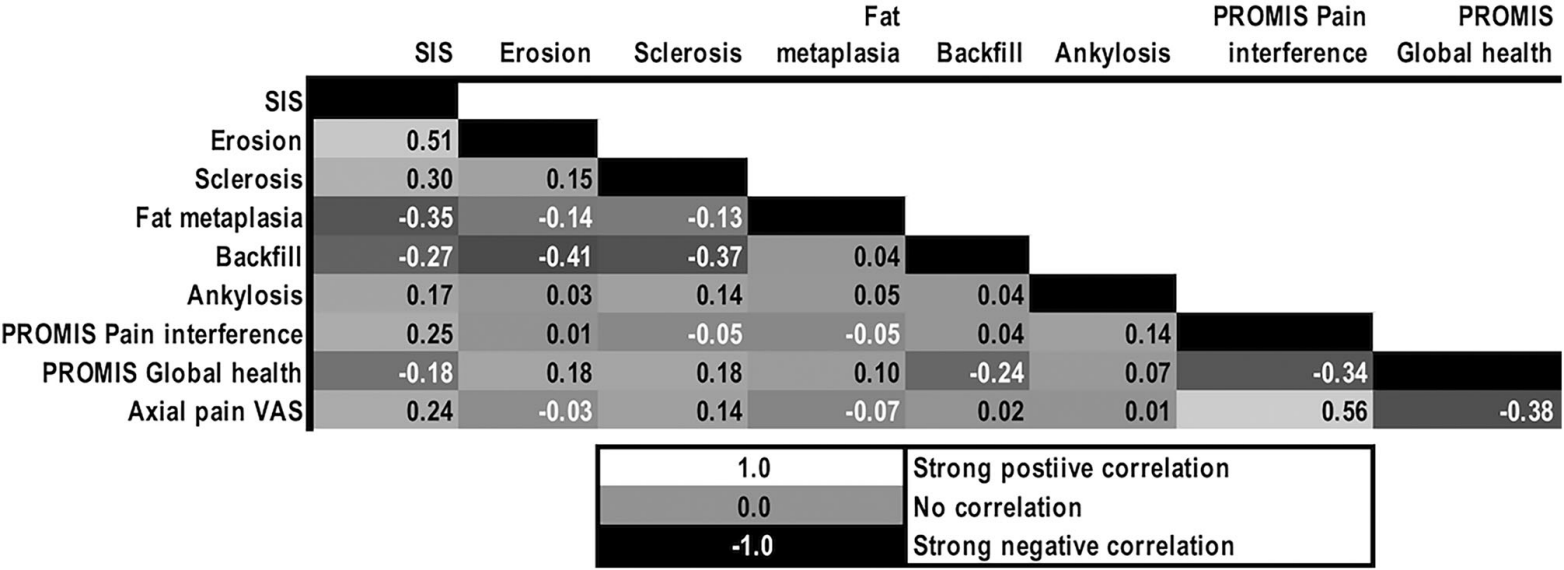
Correlation matrix of PROs and imaging outcome change scores after 12 weeks of TNFi therapy. All raw scores generated from PROMIS instruments are translated into standardized T‐scores with a population mean of 50 and SD of 10. Higher scores in a domain represent more of the trait being measured; higher T‐scores indicate a worse outcome in pain interference; lower T‐scores indicate a worse outcome global health. PRO, patient‐reported outcome; PROMIS, Patient‐Reported Outcomes Measurement Information System; SIS, sacroiliac joint inflammation score; TNFi, tumor necrosis factor inhibitor; VAS, visual analog scale.

**Figure 3:**
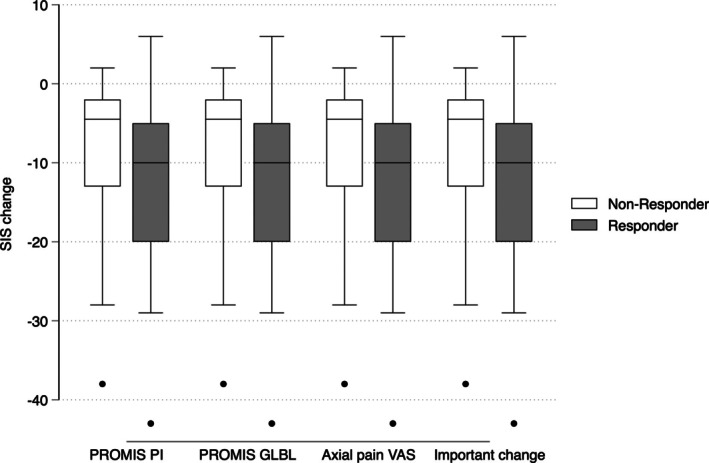
Change in SPARCC SIS by different clinical response definitions. GLBL, global health; PI, pain interference; PROMIS, Patient‐Reported Outcomes Measurement Information System; SIS, sacroiliac joint inflammation score; SpA, spondyloarthritis; SPARCC, Spondyloarthritis Research Consortium of Canada; TNFi, tumor necrosis factor inhibitor; VAS, visual analog scale.

The correlation matrix of the change in patient‐reported and imaging lesion change scores after 12 weeks of TNFi therapy is shown in Figure 2. The correlations between imaging findings and PROs were primarily low (all r less than |0.5|). A total of 84.6% (22 of 26) of those with persistent SIJ inflammation on MRI at 12 weeks reported at least moderate clinical improvement. MRI inflammation score changes by clinical responder status for PROMIS pain interference, PROMIS global health, neck/back/hip pain, and patient‐reported symptom improvement at week 12 are shown in Figure 3. Although there were no significant differences in SIS change by responder status, the median change in SIS was smaller in nonresponders than in responders for all PRO measures. There was also more variability of SIS change among responders versus nonresponders across all PROs.

## DISCUSSION

Most participants showed early imaging and clinical improvement in this prospective study of youth with incident axJSpA treated with TNFi. After 12 weeks of TNFi therapy, 89% of participants showed improvement in SIS, with a median change of −8, and 30% achieved near‐complete resolution of inflammation (SIS <2). Structural lesions were common at baseline, with erosion most frequently observed. By week 12, erosion and sclerosis scores improved in 58% and 21% of participants, respectively, although some showed worsening. Fat metaplasia and backfill, which may indicate tissue recovery, increased in approximately 30% of participants, with minimal decreases. At week 24, 44% of those with residual inflammation at week 12 (19% of the total cohort) continued to show signs of inflammation. Most imaging improvement occurred within the first 12 weeks. Despite persistent MRI inflammation, 85% reported moderate or greater clinical improvement. Correlations between imaging and PROs were generally low. These findings underscore the early impact of TNFi therapy and highlight individual variability in treatment response and imaging resolution.

This study demonstrates that although TNFi generally provides substantial relief of axial symptoms in children, not all youth have a complete imaging response. At 12 and 24 weeks, 44% and 19% of scans still showed SIJ inflammation, although most demonstrated a significant reduction. The median and mean changes in SPARCC SIS were −8 and −11.4, respectively. This observed magnitude of change is greater than the reported mean SIS changes of −3.8 and −3.2 in adults with nonradiographic axSpA treated with etanercept^17^ (Effect of Etanercept on Symptoms and Objective Inflammation in nr‐axSpA EMBARK) or adalimumab^18^ (ABILITY‐1), respectively. Greater inflammatory responses in youth may reflect shorter disease duration. In this pediatric cohort, the baseline SIS was also higher in HLA‐B27–positive participants and those with shorter symptom duration, with nonsignificant trends toward higher scores in boys, overweight participants, and those with elevated markers of inflammation. Further, there were significantly greater reductions in the SIS in boys and HLA‐B27‐positive participants. These findings highlight the importance of considering baseline disease activity and patient characteristics when designing pediatric axSpA trials.

This pediatric study revealed a more variable response to TNFi regarding SPARCC structural erosion scores compared with adult data. By week 12, erosion scores decreased (improved) in 58% of participants but increased (worsened) in 25%. In contrast, the adult EMBARK trial of patients with nonradiographic axSpA and baseline inflammation (SIS ≥2) treated with etanercept reported erosion score reductions in only 25% and increases in just 1.7%.^17^ Also, sclerosis scores decreased in 21.1% of youth in this study by week 12. Changes in sclerosis were not reported in the adult studies because this is a scored component of the SSS in youth only. The higher proportion of pediatric participants with improved erosion scores may support the “window of opportunity” hypothesis^19^ because they had a shorter duration of axial symptoms (1 of 3 of participants had a disease duration of less than 3 months versus a mean of 2.5 years in EMBARK). The greater proportion of erosion worsening (25% in youth vs 1.7% in adults) may reflect differences in age at onset, disease duration, or baseline SIJ inflammation at the time of TNFi initiation, with a mean baseline SPARCC SIS of 13.7 in youth versus 8.3 in adults. Of the 14 participants with higher erosion scores at 12 weeks, 71% had persistent inflammation on MRI (SIS ≥2).

Additionally, TNFi appeared to have a greater effect on SPARCC structural backfill and fat metaplasia scores, which are both considered potential markers of tissue repair,^19^ in this pediatric cohort compared with adults in EMBARK.^17^ By 12 weeks, backfill and fat metaplasia scores increased in 28% and 30% of participants, respectively. Only two participants showed a decrease (worsening) in the backfill score, whereas none had a reduction in the fat metaplasia score. Comparatively, in EMBARK, only 13.3% of adults treated with TNFi had an increase (improvement) in backfill scores.^17^ The greater proportion of youth with increases in these repair‐associated changes may be attributed to younger age, shorter disease duration, and more pronounced baseline SIJ inflammation at the time of TNFi initiation. Most participants in this pediatric study received adalimumab, whereas EMBARK exclusively used etanercept—another factor that may contribute to the observed differences and should be explored in future comparative studies of therapies that target distinct inflammatory pathways.

Correlations between imaging and PROs after 12 weeks of TNFi therapy were generally low (all r < |0.5|), highlighting a disconnect between clinical improvement and MRI‐detected SIJ inflammation. A total of 85% of patients (22 of 26) with persistent MRI inflammation at the 12‐week visit reported at least moderate clinical benefit. Although changes in SPARCC SIS did not significantly differ by PRO responder status, median improvements were consistently smaller in nonresponders with greater variability observed, suggesting that MRI and PROs may capture complementary aspects of treatment response, in accordance with what has previously been shown in adults.

This cohort study has several key strengths, including its multicenter design, blinded central imaging scoring, and assessment of clinical and imaging responses in a real‐world clinical context. Along with these strengths, there are limitations that should also be considered. As with any prospective, multicenter study, practice patterns likely varied across sites. To combat the differences in practice pattern, all baseline MRI scans were reviewed by a central radiologist to assess eligibility. Clinical axial disease evaluation was performed at the treating physician's discretion, and thresholds for assessment may have varied by center and provider. However, any potentially missed milder cases are unlikely to represent the patients typically considered for biologic therapy initiation or enrollment in pragmatic trials targeting axial disease. Further, each participating center has a large referral base, capturing the full spectrum of disease severity. Not all baseline MRI scans were performed with the research protocol, but all included the necessary sequences to perform quadrant‐based SPARCC scoring. All week 12 and 24 imaging studies followed the same research imaging protocol. Some loss to follow‐up occurred, primarily between weeks 12 and 24, but it was minimal.

Based on these findings, repeat imaging in clinical trials can be conducted as early as 12 weeks after initiating therapy. Tracking disease activity on MRI is important, particularly given the modest correlation with clinical measures of disease activity and function, which is why the SPARCC SIS is included in the core set of discriminating and responsive measures developed for youth with axial SpA.^20^ The data presented here provide a comparator benchmark for novel therapy trials. The decision to track lesions observed on MRI through follow‐up scans in the clinical setting should consider the patient and family's preferences, disease history, current therapy, and how the patient's axial‐related symptoms respond to treatment. Although over 80% of all inflammation in this cohort resolved after 12 weeks, 68% (13 of 19) of participants with persistent inflammation had continued improvement at week 24. In the absence of physical symptoms, repeating imaging as part of standard clinical care is likely not necessary. Future research should examine the significance of MRI findings in the absence of clinical symptoms.

In summary, TNFi therapy in youth with incident axJSpA produces rapid clinical and imaging improvement, with most gains evident within the first 12 weeks of treatment. Although residual MRI inflammation persists in a subset of patients, substantial reductions in the SIS and early structural repair changes suggest a robust TNFi treatment effect. There were notable differences in the SIS baseline and change by specific subgroups. The modest correlation between imaging and PROs underscores the complementary value of both measures in assessing the complete therapeutic response. These findings support the early use of TNFi in pediatric axJSpA and highlight the importance of considering baseline disease characteristics when designing future pediatric axJSpA trials.

## AUTHOR CONTRIBUTIONS

All authors contributed to at least one of the following manuscript preparation roles: conceptualization AND/OR methodology, software, investigation, formal analysis, data curation, visualization, and validation AND drafting or reviewing/editing the final draft. As corresponding author, Dr Weiss confirms that all authors have provided the final approval of the version to be published and takes responsibility for the affirmations regarding article submission (eg, not under consideration by another journal), the integrity of the data presented, and the statements regarding compliance with institutional review board/Declaration of Helsinki requirements.

## Supporting information


**Disclosure Form**:
